# Influence of age and dietary cellulose levels on ileal endogenous energy losses in broiler chickens

**DOI:** 10.1016/j.psj.2022.101948

**Published:** 2022-05-06

**Authors:** M.M. Khalil, M.R. Abdollahi, F. Zaefarian, P.V. Chrystal, V. Ravindran

**Affiliations:** ⁎ Monogastric Research Center, School of Agriculture and Environment, Massey University, Palmerston North, 4442, New Zealand; † Complete Feed Solutions, Howick 2145, Auckland, New Zealand

**Keywords:** broiler, age, cellulose, ileal endogenous energy loss

## Abstract

Two experiments were conducted to investigate the influence of age and dietary cellulose levels on the ileal endogenous energy losses (**IEEL**) in broiler chickens. In experiment 1, a glucose-based purified diet was used to determine the IEEL. Titanium dioxide (5.0 g/kg) was added to the diet as an indigestible marker. Six groups of broiler chickens aged 1 to 7, 8 to 14, 15 to 21, 22 to 28, 29 to 35 or 36 to 42 d posthatch, were utilized. With the exception of 1-7 d, the birds were fed a starter (d 1–21) and/or a finisher (d 22–35) diet before the experimental diet was introduced. The diet was randomly allocated to 6 replicate cages, and the number of birds per cage was 12 (d 1–7), 10 (d 8–14), and 8 (d 15–42). The ileal digesta were collected at the last day of each week (d 7, 14, 21, 28, 35, and 42). Bird age had no effect (*P* > 0.05) on the IEEL estimates. The IEEL estimates ranged from 263 to 316 kcal/kg dry matter intake (**DMI**) during weeks 1 to 6. In Experiment 2, 4 glucose-based purified diets were developed using 0, 25, 50 and 75 g/kg cellulose. Titanium dioxide (5.0 g/kg) was added to the diets as an indigestible marker. The diets were randomly allocated to 6 replicate cages (8 birds per cage) and fed from 18 to 21 d posthatch and, ileal digesta were collected on d 21. The IEEL estimates of broiler chickens at 21 d of age showed a quadratic response (*P* < 0.05) to increasing cellulose contents. The lowest IEEL (88 kcal/kg DMI) was recorded for the diet without cellulose and the highest IEEL (430 kcal/kg DMI) was observed for the diet with 75 g/kg cellulose. Overall, the present findings confirmed that the IEEL in broiler chickens can be quantified by feeding a glucose-based purified diet. Broiler age had no influence on the IEEL estimates. The IEEL increased with increasing dietary cellulose contents and the IEEL determined using a purified diet without cellulose represents a better estimate of IEEL.

## INTRODUCTION

Dietary energy is the most important aspect to be considered in diet formulations, as it represents the costliest component in poultry feeds and regulates feed intake, and the need for accurate measurement of the available energy in feed ingredients for poultry has long been recognized (Fraps, 1928). Several energy systems have been considered over the years, but the AME has become the cornerstone of poultry feed formulations since its introduction in the 1950’s (Hill and Anderson, 1958) despite suffering from several weaknesses (Mateos et al., 2019; Wu et al., 2020). The AME system is simple and can be routinely measured, and its limitations are overlooked. Apparent ileal digestible energy (**AIDE**) is an alternative measurement that has been investigated as a potential energy system (Scott et al., 1998; Camden et al., 2001; Gehring et al., 2012; Khalil et al., 2020). The AIDE is measured in the ileal digesta as a reflection of digestible nutrients instead of metabolizability measured at the excreta level. A shift to AIDE will overcome most of the weaknesses and errors associated with the AME system (Khalil et al., 2020). Importantly, the AIDE will align with the current trends in feed evaluation by measuring the digestibility of amino acids (Lemme et al., 2004), phosphorus (Mutucumarana et al., 2015), and calcium (David et al., 2021) in feed ingredients at the ileal level.

The digestible energy determined in ileal digesta is apparent and includes nondietary endogenous energy flow termed as ileal endogenous energy loss (**IEEL**). A correction for these losses enables the calculation of true ileal digestible energy (**TIDE**), which may be more additive than AIDE in feed formulations (Lemme et al., 2004; Cowieson et al., 2019). Khalil et al. (2020) reported that the TIDE showed a stronger correlation with nutrient digestibility than the AIDE. A novel approach for the quantification of IEEL in broiler chickens was developed and proposed in our previous study (Khalil et al., 2020). Feeding a glucose-based purified diet was proved to be an acceptable method for the estimation of IEEL, as the glucose was completely absorbed before the lower ileum, indicating that the energy determined from the digesta collected at the lower ileum could have originated only from nondietary components.

The IEEL may be affected by factors similar to those affecting endogenous amino acid losses (Adedokun et al., 2011; Adeola et al., 2016; Ravindran, 2021). Age of birds has been reported to influence the endogenous energy losses determined in the excreta (Murakami et al., 1995). Silva et al. (2011) observed that the endogenous and metabolic energy losses increased linearly with the advancing age of broiler chickens. However, all previous studies have determined the endogenous energy losses in the excreta (Dale and Fuller, 1982; Sibbald, 1982; Pirgozliev et al., 2009), and, to the authors’ knowledge, there is no published report investigating the effect of broiler age on the IEEL.

In our previous study (Khalil et al., 2020), to ensure the diet texture and uniform digesta passage in the digestive tract of the birds, 50 g/kg of cellulose was included in the glucose-based purified diet. Cellulose is not digested by birds and would have contributed to the undigested components in the terminal ileum, resulting in an overestimation of the IEEL. Therefore, the objectives of the current study were 2-fold. First to investigate whether the age of broilers influences IEEL estimates and second to examine whether inclusion levels of dietary cellulose influence the IEEL.

## MATERIALS AND METHODS

The experiments were conducted according to the New Zealand Revised Code of Ethical Conduct for the use of live animals for research, testing and teaching and approved by the Massey University Animal Ethics Committee.

### Diets, Birds and Housing

For experiment 1, a glucose-based purified diet, containing 900 g/kg glucose, was developed (Table 1). Titanium dioxide (**Ti**) was included in the diet as an indigestible marker at an inclusion rate of 5.0 g/kg.

**Table 1:** Composition of the glucose-based purified diet^1^ (g/kg, as fed basis), Experiment 1.

Ingredients	Inclusion, g/kg
Glucose^2^	900
Cellulose^3^	50
Dicalcium phosphate	20
Limestone	13
Titanium dioxide	5.0
Sodium bicarbonate	3.0
Sodium chloride	3.0
Dipotassium phosphate	1.0
Vitamin-trace mineral premix^4^	5.0

1 Analyzed calcium content,10.4 g/kg; phosphorus content, 4.21 g/kg.

2 Glucose, Dexmonc, Davis food ingredients, Victoria, Australia.

3 Solkafloc, Ceolus PH-102, Asahi Kasei Corporation, Tokyo, Japan. Added to maintain uniform passage and consistency of digesta in the digestive tract.

4 Vitamin and trace mineral premix supplied the following per kilogram of diet: antioxidant, 100 mg; biotin, 0.2 mg; calcium pantothenate, 12.8 mg; vitamin D_3_ (cholecalciferol), 2400 IU; cyanocobalamin, 0.017 mg; folic acid, 5.2 mg; menadione, 4 mg; niacin, 35 mg; pyridoxine, 10 mg; vitamin A (trans-retinol), 11100 IU; riboflavin, 12 mg; thiamine, 3.0 mg; vitamin E (dl-α-tocopheryl acetate), 60 IU; choline chloride, 638 mg; Co, 0.3 mg; Cu, 3.0 mg; Fe, 25 mg; I, 1 mg; Mn, 125 mg; Mo, 0.5 mg; Se, 200 µg; Zn, 60 mg.

A total number of 324, day-old male broilers (Ross 308) were obtained from a local hatchery and raised on floor pens. Except for the 1 to 7 d age group, birds were fed broiler starter pellets (Table 2; 225 g/kg crude protein and 2,900 kcal/kg AME) until d 21 and finisher pellets (Table 2; 190 g/kg crude protein and 3,030 kcal/kg AME) from d 22 to 35 before they switched to the assay diet (Table 1). At the beginning of each week (d 1, 8, 15, 22, 29, and 36), birds were selected randomly from floor pens, individually weighed, and allocated to 6 replicate cages during 6 periods, namely week 1 (d 1–7), week 2 (d 8–14), week 3 (d 15–21), week 4 (d 22–28), week 5 (d 29–35), or week 6 (d 36–42). Each replicate cage housed 12 birds during week 1, 10 birds during week 2, and 8 birds during weeks 3 to 6 posthatch. For each age period, birds were offered the starter or finisher diet in mash form for the first 4 d as the purified diet was in mash form. The glucose-based purified diet was offered ad libitum for the last 3 d in each week.

**Table 2:** Composition (g/kg, as fed basis) of the broiler starter (d 1 to 21) and finisher (d 22 to 35) diets, Experiment 1.

Ingredient	Starter diet	Finisher diet
Corn	574.2	660.0
Soybean meal, 460 g/kg	381.4	295.6
Soybean oil	8.8	13.6
Dicalcium phosphate	10.7	8.2
Limestone	11.3	9.9
L Lysine HCl	2.0	1.9
DL Methionine	3.3	3.0
L Threonine	1.0	0.7
Sodium chloride	2.5	2.5
Sodium bicarbonate	2.7	2.5
Trace mineral premix^1^	1.0	1.0
Vitamin premix^1^	1.0	1.0
Ronozyme HiPhos (Phytase)	0.1	0.1
Calculated analysis		
AME (kcal/kg)	2,900	3,030
CP	225	190
Digestible lysine	11.0	9.2
Digestible methionine	6.2	5.6
Digestible methionine + cysteine	9.2	8.3
Digestible threonine	7.2	6.0
Crude fat	32	39
Crude fiber	29.3	27.5
Calcium	9.8	8.5
Available phosphorus	4.9	4.2
Sodium	2.2	2.1
Chloride	2.3	2.3
Potassium	11.5	9.7

1 Vitamin and trace mineral premix supplied the following per kilogram of diet: antioxidant, 100 mg; biotin, 0.2 mg; calcium pantothenate, 12.8 mg; vitamin D_3_ (cholecalciferol), 2400 IU; cyanocobalamin, 0.017 mg; folic acid, 5.2 mg; menadione, 4 mg; niacin, 35 mg; pyridoxine, 10 mg; vitamin A (trans-retinol), 11100 IU; riboflavin, 12 mg; thiamine, 3.0 mg; vitamin E (dl-α-tocopheryl acetate), 60 IU; choline chloride, 638 mg; Co, 0.3 mg; Cu, 3.0 mg; Fe, 25 mg; I, 1 mg; Mn, 125 mg; Mo, 0.5 mg; Se, 200 µg; Zn, 60 mg.

Experiment 2 was initiated to determine the effect of dietary cellulose inclusion levels in a glucose-based purified diet on the IEEL estimates in broiler chickens. Four assay diets were developed using 0, 25, 50, or 75 g/kg cellulose at the expense of glucose (Table 3). Diets were mixed individually in a single-screw paddle mixer (Bonser Engineering Co. Pty. Ltd., Merrylands, Australia). Titanium dioxide was included in the diet as an indigestible marker at an inclusion rate of 5.0 g/kg.

**Table 3:** Composition of the glucose-based purified diets^1^ (g/kg, as fed basis), Experiment 2.

Ingredients	Cellulose content (g/kg)			
	0	25	50	75
Glucose^2^	950	925	900	875
Cellulose^3^	0.0	25	50	75
Dicalcium phosphate	20	20	20	20
Limestone	13	13	13	13
Titanium dioxide	5.0	5.0	5.0	5.0
Sodium bicarbonate	3.0	3.0	3.0	3.0
Sodium chloride	3.0	3.0	3.0	3.0
Dipotassium phosphate	1.0	1.0	1.0	1.0
Vitamin-trace mineral premix^4^	5.0	5.0	5.0	5.0

1 Analyzed calcium content, 10.4 g/kg; phosphorus content, 4.21 g/kg.

2 Glucose, Dexmonc, Davis food ingredients, Victoria, Australia.

3 Solkafloc, Ceolus PH-102, Asahi Kasei Corporation, Tokyo, Japan.

4 Supplied per kg diet: antioxidant, 100 mg; biotin, 0.2 mg; calcium pantothenate, 12.8 mg; cholecalciferol, 60 µg; cyanocobalamin, 0.017 mg; folic acid, 5.2 mg; menadione, 4 mg; niacin, 35 mg; pyridoxine, 10 mg; trans-retinol, 3.33 mg; riboflavin, 12 mg; thiamine, 3.0 mg; dl-α-tocopheryl acetate, 60 mg; choline chloride, 638 mg; Co, 0.3 mg; Cu, 3.0 mg; Fe, 25 mg; I, 1 mg; Mn, 125 mg; Mo, 0.5 mg; Se, 200 µg; Zn, 60 mg.

A total number of 144, day-old male broilers (Ross 308) were obtained from a local hatchery and raised on floor pens. Birds were fed broiler starter pellets (225 g/kg crude protein and 2,900 kcal/kg AME) until d 18 when they switched to assay diets. On d 14, birds were weighed individually, allocated to cages and offered the starter diet in mash form from d 14 to 17, as the purified diets were in mash form. The assay diets were then offered from d 18 to 21. Each assay diet was fed to 6 replicate cages (6 birds per cage).

In both experiments, the floor pens and cages were housed in environmentally controlled rooms with 20 h of fluorescent illumination per day, and feed and water were offered ad libitum. The temperature was maintained at 31°C on day 1 and was gradually reduced to 22°C by the end of the third week. Central ceiling extraction fans and wall inlet ducts-controlled ventilation.

### Ileal Digesta Collection

At the final day of each week (d 7, 14, 21, 28, 35, and 42) in experiment 1 and d 21 in experiment 2, all birds in a cage were euthanized by intravenous injection (1 mL per 2 kg live weight) of sodium pentobarbitone (Provet NZ Pty Ltd., Auckland, New Zealand). The small intestine was isolated and the digesta from the terminal ileum were collected. The ileum was defined as the portion of the small intestine extending from Meckel's diverticulum to ∼40 mm proximal to ileo-cecal junction and the ileal digesta were collected from the lower half towards the ileo-cecal junction. The digesta were removed by gentle flushing with distilled water, as described by Ravindran et al. (2005). Digesta were pooled within a cage, lyophilized (Model 0610, Cuddon Engineering, Blenheim, New Zealand), ground to pass through a 0.5-mm sieve and stored at 4°C until laboratory analysis. The feed and digesta samples were analyzed for DM, gross energy (**GE**), glucose and Ti.

### Chemical Analysis

Dry matter was determined using standard procedures (Methods 930.15; AOAC, 2016). Samples were assayed for Ti on a UV spectrophotometer following the method of Short et al. (1996). Glucose was determined using an assay kit (Rx Daytona Plus, Randox Laboratories Ltd, Crumlin, UK) following enzymatic oxidation in the presence of glucose oxidase. Gross energy was determined by an adiabatic bomb calorimeter (Gallenkamp Autobomb, Weiss Gallenkamp Ltd, Loughborough, UK) standardized with benzoic acid.

### Calculations

Apparent ileal absorption of glucose was calculated using the Ti ratios in the diet and digesta as shown below. All concentrations were expressed as g per kg DM.

$$\text{Apparent}\;\text{absorption}=1-\left[\left(Ti_{\text{Diet}}/Ti_{\text{Digesta}}\right)\times \left(\text{Glucos}e_{\text{Digesta}}/\text{Glucos}e_{\text{Diet}}\right)\right]$$

The flow of ileal endogenous energy, as kcal lost per kilogram of DM intake (**DMI**), was calculated by using the following formula:

$$\text{Endogenous}\;\text{energy}\;\text{losses}\;\left(\text{kcal}/\text{kg}\;\text{DMI}\right)=GE_{\text{Digesta}}\left(\text{kcal}/\text{kg}\right)\times \left[Ti_{\text{Diet}}\left(g/\text{kg}\right)/Ti_{\text{Digesta}}\left(g/\text{kg}\right)\right]$$

Apparent ileal digestibility data for GE were then converted to true digestibility values, using IEEL determined from birds fed the glucose-based purified diet without cellulose.

$$\begin{array}{c} \text{Coefficient}\;\text{of}\;\text{true}\;\text{ileal}\;\text{energy}\;\text{digestibility}=\\ \text{CAID}\;\text{of}\;\text{GE}+\left[\text{Basal}\;\text{IEEL}\;\left(\text{kcal}/\text{kg}\;\text{DMI}\right)/GE_{\text{Diet}}\left(\text{kcal}/\text{kg}\right)\right] \end{array}$$

$$\text{TID}E_{\text{Diet}}=\text{Coefficient}\;\text{of}\;\text{true}\;\text{ileal}\;\text{energy}\;\text{digestibility}\times GE_{\text{Diet}}$$

### Statistical Analysis

The data were analyzed as a one-way ANOVA using the General Linear Model procedure of SAS (version 9.4; SAS Institute Inc., Cary, NC). Cage served as the experimental unit. Significant differences between means were separated by Least Significant Difference test. In addition, the data were subjected to orthogonal polynomial contrasts using the General Linear Models procedure of SAS (2015) to study whether responses to increasing bird age (experiment 1) or cellulose level (experiment 2) were of linear or quadratic nature. Significance of effects was declared at *P* ≤ 0.05.

## RESULTS

The estimates for the IEEL and coefficient of apparent glucose absorption in the ileum are presented in Table 4. The IEEL was unaffected (*P* > 0.05) by bird age. During weeks 2 to 6, the coefficient of apparent glucose absorption was determined to be 1.00, confirming complete glucose absorption by the time digesta reaches terminal ileum. There was insufficient digesta samples at week 1 for glucose analysis.

**Table 4:** Influence of age on the ileal endogenous energy loss (kcal/kg dry matter intake) and coefficient of apparent glucose absorption in broilers fed a glucose-based purified diet, Experiment 1.^1^

Age (week)	Endogenous energy loss	Apparent glucose absorption coefficient^2^
1	315	-
2	280	1.00
3	277	1.00
4	292	1.00
5	263	1.00
6	284	1.00
SEM^3^	13.6	0.001
*P*-value	0.193	0.426
Orthogonal polynomial contrast, *P* ≤		
Linear	0.104	-
Quadratic	0.162	-

1 Each value represents the mean of 6 replicates. The number of birds per replicate cage were 12 (week 1), 10 (week 2) and 8 (weeks 3–6).

2 Analyzed glucose values: diet, 892 g/kg; ileal digesta, week 1, insufficient sample size; week 2, 2.01 ± 1.69; week 3, 0.43 ± 0.11; week 4, 0.56 ± 0.26; week 5, 0.28 ± 0.08; week 6, 2.35 ± 0.94 g/kg (mean ± SD; 6 replicates).

3 Pooled standard error of mean.

The estimates for the IEEL and coefficient of apparent glucose absorption in the ileum of birds fed glucose-based purified diets with different inclusion levels of cellulose are presented in Table 5. The IEEL increased (*P* < 0.01) with increasing dietary cellulose inclusions and the magnitude of responses differed between inclusion levels, resulting in a quadratic effect (*P* < 0.05). The lowest IEEL (88 kcal/kg DMI) was recorded for the diet without cellulose and the highest (430 kcal/kg DMI) for the diet with 75 g/kg cellulose. Complete glucose absorption was determined at the ileal level, regardless of dietary cellulose content.

**Table 5:** Influence of dietary cellulose content on the ileal endogenous energy loss (kcal/kg dry matter intake) and coefficient of apparent glucose absorption in broilers fed glucose-based purified diets, Experiment 2.^1^

Cellulose content (g/kg)	Endogenous energy loss	Apparent glucose absorption coefficient^2^
0	88^d^	1.00
25	182^c^	1.00
50	289^b^	1.00
75	430^a^	1.00
SEM^3^	9.3	0.001
*P*-value	0.001	0.254
Orthogonal polynomial contrast, *P* ≤		
Linear	0.001	-
Quadratic	0.015	-

Means in a column not sharing a common letter (a-d) are significantly different (*P* < 0.05).

1 Each value represents the mean of 6 replicates (8 birds per replicate).

2 Analyzed glucose values: No cellulose diet, 942 g/kg; 25 g/kg cellulose diet, 917 g/kg; 50 g/kg cellulose diet, 892 g/kg; 75 g/kg cellulose diet, 867 g/kg; ileal digesta for No cellulose diet, 3.04 ± 3.28; 25 g/kg cellulose diet, 4.58 ± 5.52; 50 g/kg cellulose diet, 0.85 ± 0.17; 75 g/kg cellulose diet, 0.79 ± 0.22 g/kg (mean ± SD; 6 replicates).

3 Pooled standard error of mean.

## DISCUSSION

The methodology for the determination of IEEL by feeding broiler chickens with a glucose-based purified diet in our previous study (Khalil et al., 2020) was validated in the current study. The previous estimate of IEEL (347 kcal/kg DMI) was determined using 21 d old broiler chickens and used to correct the AIDE values to TIDE in birds with the same age (Khalil et al., 2020). However, the application of a single IEEL estimate as a correction factor for different broiler ages may be challenged. A recent study in our laboratory (Barua et al., 2021) revealed that the basal ileal endogenous amino acid flow was influenced by broiler age and that the flows decreased quadratically with age with values being higher on d 7, decreasing on d 14, plateauing until d 35 and decreasing further on d 42. It was plausible that the IEEL in broilers may also vary with age and, therefore, an objective of the present work was to investigate the effect of bird age on IEEL estimates. The current findings, however, showed that bird age had no effect on IEEL estimates. Similar to our previous findings (Khalil et al., 2020) and regardless of bird age, glucose completely disappeared at terminal ileal level, suggesting that the dietary glucose was completely absorbed by the time the digesta reached terminal ileum. Riesenfeld et al. (1980), in a study with a glucose-based diet, reported complete absorption of glucose in the lower ileum. These findings could be explained by the fact that glucose, a simple monosaccharide, is the end-product of carbohydrate digestion and completely absorbed from the intestine without the need for enzymatic digestion (Herman, 1974). Therefore, glucose absorption is not influenced by age-related developments and maturation of the intestinal tract, and that it is well absorbed in the newly hatched chick (Moran et al., 2010; Ravindran and Abdollahi, 2021). In agreement, Bogner and Haines (1964) stated that the maximum absorption of glucose occurred during the first week post-hatch, with minor changes in the following weeks. Obst and Diamond (1992) reported that the absorption of glucose in the intestine was constant from 1 to 84 d of age in broiler chickens.

In Experiment 1, the IEEL estimates in broilers of 1 to 6 wk age ranged from 263 to 316 kcal/kg DMI. The IEEL value of 277 kcal/kg DMI at 3 wk of age was substantially lower than the IEEL of 347 kcal/kg DMI determined in broilers of similar age in our previous research (Khalil et al., 2020). No comparable data on IEEL in poultry are available in the literature. All previous studies estimating the endogenous energy losses in poultry were determined in the excreta of broilers or adult roosters (Sibbald, 1982; Murakami et al., 1995; Silva et al., 2011). Sibbald (1981) estimated the endogenous energy losses in fasted adult birds and found that the metabolic plus endogenous energy losses increased by 14.3% between weeks 19 and 22 of age. Similarly, Murakami et al. (1995) showed that the endogenous energy losses increased with advancing age of broiler chickens that was attributed to increasing dietary energy consumption. Silva et al. (2011) reported that the endogenous and metabolic energy losses increased linearly with advancing age of broilers from 20.87 kcal/bird at d 5 to 76.54 kcal/bird at d 35 posthatch. These endogenous energy losses estimated at the excreta level include both metabolic and endogenous losses in the urine and feces, and therefore cannot be compared with the IEEL estimates from the current study. Moreover, the metabolic and endogenous losses were expressed as a function of time (kcal/bird/d) rather than as per DMI.

In studies with purified diets, a source of fiber, usually cellulose, is included as a structural component and to texturize the feed. Therefore, cellulose was included at 50 g/kg in the glucose-based purified diet in our earlier study (Khalil et al., 2020) and Experiment 1 in this study. Cellulose is an insoluble fiber material composed of a linear chain of β-1,4-linked D-glucopyranosyl residues (O'Sullivan, 1997; Gilbert, 2010). Cellulose is inert and has no nutritional value for poultry, but typically included in purified diets for its physiological role as a bulking agent to enable a steady and uniform digesta passage rate (Siri et al., 1992). Cellulose being indigestible would have contributed to the gross energy determined from undigested materials in the ileum, resulting in overestimation of IEEL values reported by Khalil et al. (2020) and in Experiment 1. Therefore, to fine-tune the methodology for the quantification of IEEL, Experiment 2 was designed to investigate whether the IEEL estimates are impacted by the inclusion levels of cellulose in the glucose-based purified diet. The current data clearly demonstrated the impact of cellulose inclusion level on IEEL estimates, with increasing the inclusion of cellulose from 0 to 25, 50 and 75 g/kg increased the IEEL estimates by 93, 201 and 342 kcal/kg DMI, respectively. The GE content of cellulose used in the current study was 3,848 kcal/kg, which was similar to the GE value of 4,111 kcal/kg for cellulose powder reported by Kienzle et al. (2001). Tasaki and Kibe (1959) found that cellulose was excreted almost completely undigested when birds fed a basal diet supplemented with 200 g/kg cellulose. Siri et al. (1992) reported that increasing the dietary cellulose content from 50 to 100 g/kg increased the excreta energy output by 50%. As cellulose is not digested by the bird, it can contribute to the undigested matter in the ileal digesta, resulting in an overestimation of the IEEL.

The endogenous materials are derived mainly from proteins, including desquamated epithelial cells, gastrointestinal secretions (bile, gastric, pancreatic, and intestinal secretions), and mucoproteins. The increase in the IEEL associated with increasing the cellulose inclusions could be related to 2 possible reasons; first, the increase in the mechanical damage to the absorptive surface of the intestinal epithelial cell wall caused by greater inclusions of cellulose (Hegde et al., 1982). Okumura et al. (1982) found that the excretion of nitrogen increased with increasing dietary cellulose levels from 0 to 50 g/kg. A similar influence of cellulose on ileal endogenous amino acid losses was reported in a study by Kluth and Rodehutscord (2009). These researchers reported that the ileal endogenous losses of amino acids increased by 29% when the dietary cellulose level increased from 30 to 80 g/kg. Second, cellulose is reported to increase gastrointestinal secretions, mainly mucin. Mucin is the main glycoprotein of the mucus layer secreted by the goblet cells and, plays a major role in protecting the gut from physical, chemical and enzymatic damages, along with removal of pathogenic bacteria (Sharma and Schumacher, 1995; Satchithanandam et al., 1996; Montagne et al., 2003). The secretion of mucin is altered by several factors, including dietary fiber and physical properties. Several studies have demonstrated that dietary fiber increased mucin secretion (Fuller and Cadenhead, 1991; Mariscal-Landin et al., 1995; Lien et al., 2001). Montagne et al. (2003) stated that insoluble dietary fiber is more aggressive in scraping the mucin from gut wall as it passes through the digestive tract. In addition, Jha and Mishra (2021) speculated that dietary fiber may increase the stimulation and secretion of endogenous digestive enzymes in broilers.

Aderibigbe et al. (2021) calculated the IEEL originating from endogenous amino acids in broilers following the feeding of a nitrogen free diet to be around 57 to 60 kcal/kg DMI, which was lower than the IEEL estimate (88 kcal/kg DMI) in the diet containing no cellulose in the current study. The higher IEEL estimate in the current study could be related to the contribution of nonprotein components to the IEEL.

The TIDE was proposed in our previous study (Khalil et al., 2020) as a potential energy system in poultry feed formulation as it not only overcomes the limitations of the AME but also aligns energy availability with the current trend of using digestible nutrient contents in feed formulations (Lemme et al., 2004; Mutucumarana et al., 2015; David et al., 2021). Moreover, the findings from our previous study (Khalil et al., 2020) showed that, compared to the AME and AMEn of cereal grains, TIDE was highly correlated with the coefficient of apparent ileal digestibility of DM, starch, and N.

The prime application of the quantified IEEL is for the correction of AIDE to TIDE, and therefore, accurate estimation of the IEEL is needed for calculation of the TIDE value of feed ingredients. Two specific concerns regarding IEEL were raised from the previous research (Khalil et al., 2020) namely, the use of a single IEEL value for broilers of different ages and the overestimation of IEEL due to the inclusion of cellulose in the purified test diet. The current data showed that age had no influence on IEEL estimates. The lack of age effect on IEEL estimates suggest that a single basal IEEL value could be used for correction of AIDE to TIDE across broiler ages. However, dietary cellulose inclusion had a marked impact on IEEL estimates. In Khalil et al. (2020), the TIDE values of cereal grains were calculated using the IEEL value of 347 kcal/kg DMI, determined following the feeding of glucose-based purified diet with 50 g/kg cellulose. The resultant TIDE values were higher than their counterpart AIDE, AME, and AMEn values for all cereal grains. Using the AIDE values of the cereal grains from our earlier research (Khalil et al., 2020), the TIDE of cereal grains was re-calculated using the IEEL value of 88 kcal/kg DMI, determined by feeding the glucose-based purified diet without cellulose (Table 6). Although the re-calculated TIDE values were smaller than those reported in Khalil et al. (2020), and no significant differences were observed between AME, AMEn, AIDE, and TIDE for all cereal grains, the TIDE values tended to be greater in wheat (*P* = 0.053), barley (*P* = 0.111), and corn (*P* = 0.077).

**Table 6:** Apparent metabolizable energy (AME; kcal/kg DM),^1^ nitrogen-corrected AME (AMEn; kcal/kg DM)^1^ and apparent ileal digestible energy (AIDE; kcal/kg DM)^1^ and true ileal digestible energy (TIDE; kcal/kg DM)^2^ in different cereal grains in broilers at 21 d of age.

Method	Wheat	Sorghum	Barley	Corn
AME	2,653	3,346	2,447	3,499
AMEn	2,576	3,284	2,371	3,439
AIDE	2,758	3,296	2,519	3,544
TIDE	2,882	3,394	2,629	3,647
Probabilities, *P* ≤	0.053	0.442	0.111	0.077
SEM^3^	76	52	73	54

1 Each value represents the mean of 6 replicates (8 birds per replicate). Values are from Khalil et al. (2020).

2 Apparent ileal digestible energy values were corrected to true ileal digestible energy using the ileal endogenous energy flow of 88 kcal/kg DM intake, determined by feeding glucose-based diet without cellulose.

3 Pooled standard error of mean.

## CONCLUSIONS

The present study provides, for the first time, data on the IEEL in broiler chickens from 1 to 6 wk of age. The findings confirm that the IEEL in broiler chickens can be quantified using glucose-based purified diet and that the age of birds has no impact on the IEEL. The dietary cellulose content had substantial impact on IEEL estimates and it is suggested that the IEEL determined using a purified diet with no added cellulose represents a better estimate. Some aspects relevant to the determination of TIDE were explored in the studies reported in the current work and our previous study, but further research is warranted before TIDE could be adopted as an energy system in poultry feed formulations. In particular, well-planned feeding trials comparing formulations based on metabolisable energy versus ileal digestible energy and, their impact on broiler growth performance and the production economics will be instructive.
